# Amyloid-β-Acetylcholinesterase complexes potentiate neurodegenerative changes induced by the Aβ peptide. Implications for the pathogenesis of Alzheimer's disease

**DOI:** 10.1186/1750-1326-5-4

**Published:** 2010-01-18

**Authors:** Margarita C Dinamarca, Juan P Sagal, Rodrigo A Quintanilla, Juan A Godoy, Macarena S Arrázola, Nibaldo C Inestrosa

**Affiliations:** 1 Centro de Regulación Celular y Patología "Joaquín V. Luco" (CRCP), Instituto Milenio MIFAB, Centro de Envejecimiento y Regeneración (CARE), Facultad de Ciencias Biológicas, Pontificia Universidad Católica de Chile, Alameda 340, 8331010 Santiago, Chile

## Abstract

The presence of amyloid-β (Aβ) deposits in selected brain regions is a hallmark of Alzheimer's disease (AD). The amyloid deposits have "chaperone molecules" which play critical roles in amyloid formation and toxicity. We report here that treatment of rat hippocampal neurons with Aβ-acetylcholinesterase (Aβ-AChE) complexes induced neurite network dystrophia and apoptosis. Moreover, the Aβ-AChE complexes induced a sustained increase in intracellular Ca^2+ ^as well as a loss of mitochondrial membrane potential. The Aβ-AChE oligomers complex also induced higher alteration of Ca^2+ ^homeostasis compared with Aβ-AChE fibrillar complexes. These alterations in calcium homeostasis were reversed when the neurons were treated previously with lithium, a GSK-3β inhibitor; *Wnt-7a *ligand, an activator for *Wnt *Pathway; and an N-methyl-D-aspartate (NMDA) receptor antagonist (MK-801), demonstrating protective roles for activation of the *Wnt *signaling pathway as well as for NMDA-receptor inhibition. Our results indicate that the Aβ-AChE complexes enhance Aβ-dependent deregulation of intracellular Ca^2+ ^as well as mitochondrial dysfunction in hippocampal neurons, triggering an enhanced damage than Aβ alone. From a therapeutic point of view, activation of the *Wnt *signaling pathway, as well as NMDAR inhibition may be important factors to protect neurons under Aβ-AChE attack.

## Background

Alzheimer's disease (AD) is an age-related neurodegenerative disease characterized by selective neuronal cell death that affects brain areas related to memory and learning [1]. The neuropathological hallmarks of AD patients are the presence of senile plaques and neurofibrillary tangles in the brain [2]. Senile plaques are aggregates of deposited amyloid-β peptide (Aβ), surrounded by dystrophic neurites and reactive glial cells [2]. Aβ-peptide is the main constituent of senile plaques and a major neurotoxic agent [2]. Other proteins associated to amyloid deposits, known as "chaperone molecules" [3] include laminin, apolipoprotein E and acetylcholinesterase (AChE) [3-5]. In fact, AChE has been found to co-localize with Aβ deposits such at those present in pre-amyloid diffuse deposits, mature senile plaques and cerebral blood vessels [6,7]. Most of the cortical AChE activity present in AD brain is predominantly associated to the amyloid core of senile plaques rather than with the neuritic component found in the periphery [7]. More than 10 years ago, we found that AChE a key enzyme in the degradation of the neurotransmitter acetylcholine, present in cholinergic terminals accelerates Aβ aggregation [4], promoting the formation of a stable complex with the enzyme (Aβ-AChE complex) [8]. We showed for the first time that a macromolecule found in the synapse interacts with Aβ to form a complex which alters the normal synaptic function in hippocampal neurons. *In vivo *studies showed that AChE infused stereotaxically into the CA1 region of the rat hippocampus promotes novel plaque-like structures [9,10]. More recently, independent studies support our initial observation indicating that AChE accelerates Aβ deposition, in fact a double transgenic mouse over expressing both the human APP containing the Swedish mutation and the human AChE has been developed. Such double transgenic mice start to form amyloid plaques around 3 months, earlier than mice expressing only the APP transgene. Moreover, the double AChE-APP transgenic mouse presents more and larger plaques than the control animals, as well as some behavioural deterioration, as demonstrated by a working memory test [11]. Indeed, injection of the complex into the rat hippocampus produces neuronal cell loss and astrocyte hypertrophy [10].

The early events triggered in neurons in response to Aβ peptide have been largely studied [12-16]. It has been described that Aβ oligomers/fibrils induce intracellular calcium deregulation that leads to apoptosis through mitochondria dysfunction, whether by direct interaction with isolated mitochondria or by indirect association with the neuronal membrane [12-16]. We report here the early effects that Aβ-AChE complexes induce in rat hippocampal neurons using live-cell imaging techniques. Results show that Aβ-AChE complexes are more toxic than the Aβ fibrils alone on rat hippocampal neurons. In fact, neurons treated with Aβ-AChE complexes showed a much disrupted neurite network compared to neurons treated with Aβ. One the earliest effect of Aβ-AChE complexes is an increase in intracellular calcium, which leads to the loss of the mitochondrial membrane potential, this being in agreement with the notion that calcium homeostasis and mitochondrial function are the main targets of these complexes.

## Results

### Aβ-AChE complexes disrupt neuronal morphology and induce intracellular calcium increase in hippocampal neurons

**Figure 1:**
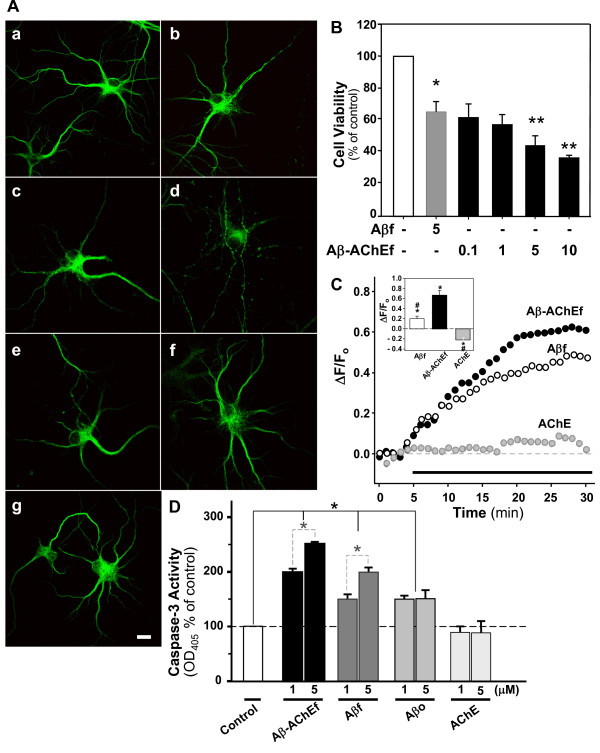
**Aβ and Aβ-AChE complexes induce morphological changes, apoptosis and intracellular Ca^2+ ^increase in rat hippocampal neurons**. (A) Immunofluorescence for MAP-2 protein of 14 DIV hippocampal neurons treated with: (a) control, (b) 5 μM Aβ-AChEo, (c) 5 μM Aβo, (d) 5 μM Aβ-AChEf, (e) 5 μM Aβf, (f) 5 nM AChE and (g) 5 μM Aβ_42-1 _for 1 h. Scale bar, 10 μm. (B) Cell viability was performed with the MTT reduction assay in hippocampal neurons treated for 12 h with the indicated μM concentrations. *p ≤ 0.01 and **p ≤ 0.001 compared with the control condition. (C) Hippoccampal neurons in culture were loaded with Fluo-3 AM (5 μM for 30 min at 37°C) to measure changes in free intracellular Ca^2+^. The graph shows normalized fluorescence intensities according to the ratio ΔF/F_o _(arbitrary units) in function of time. Black bar indicates onset of treatment. 5 μM Aβf (White circle); 5 μM Aβ-AChEf (Black circle); 5 nM AChE (Grey circle). Inset shows the final normalized fluorescence intensities reached at the end of 1 h of recording. *p ≤ 0.001 compared to control; #*p *≤ 0.001 compared to the Aβ-AChEf treatment. (D) Detection of Caspase-3 activity in hippocampal neurons. The graph shows the activity of Caspase-3 under effect of different preparation of Aβ and Aβ-AChE peptide. The cells were treated by 1 h at 37°C. Results are the mean ± S.E.M; *p < 0.05.

In order to evaluate the morphological changes induced by Aβ-AChE complexes in hippocampal neurons, the following immunofluorescence studies were performed. Hippocampal neurons were treated with 5 μM of Aβ preparations: Aβ fibrils (Aβf), Aβ oligomers (Aβo), Aβ-AChE mostly fibers (Aβ-AChEf) and Aβ-AChE mostly oligomers (Aβ-AChEo) (see additional file 1). We used an Aβ reverse sequence (5 μM) and AChE (5 nM), for 1 hr as controls. Neurons were stained for MAP-1B (Fig. 1A) or NF-200 (Table 1). Neurons treated with Aβo (Fig. 1Ac) were observed to have a higher loss of their neurite network compared to neurons treated with Aβf (fig 1Ae). Furthermore, while neurons treated with Aβ-AChEo (Fig. 1Ab) appeared to have a small, loss of their neuritic network, neurons treated with Aβ-AChEf (Fig. 1Ad) were observed to have more damage on their neurite network than neurons treated with Aβf (Fig. 1Ae). Complementary immunofluorescence studies with a NF-200 antibody showed that Aβ-AChEf significantly decreased the length of the neurites by 40%, in comparison with Aβf-treated neurons (see Table 1). Additionally, we evaluated the effect of different Aβ and Aβ-AChE preparations on synaptic proteins (Additional file 2). We performed an immunofluorescence assay for presynaptic protein synapsin-1 and postsynaptic protein PSD-95. In agreement with previous studies, Aβo treatment decreased the immunostaining for PSD-95 whereas Aβf had no effect [17]. Also, Aβ-AChEo treatment induced a critical decrease in PSD-95 immunofluorescence similar to Aβo preparations, whereas Aβ-AChEf had no effect (Additional file 2), suggesting that Aβ oligomers formed in the presence of AChE have a similar synaptotoxicity to those formed in its absence.

**Table 1:** Aβ-AChE complex induces a neurite network loss in hippocampal neurons

Treatment	Neurite length (μm)
Control	65 ± 7

Aβ (5 μM)	30 ± 4*

Aβ-AChE (5 μM)	28 ± 5*

Determinations are representative of 3 different experiments carried out. Anti-NF-200 antibody was used for the immunofluorescence analysis. Asterisk indicated statistical difference between Control and Aβ (*) p < 0.001, and Control and Aβ-AChE (*) p < 0.001.

To determine whether Aβ-AChE is more toxic than Aβ, we performed cell viability studies using MTT, as an indicator of cell viability. Aβf alone decreased cell viability by 35 ± 7% when compared with control, while Aβ-AChEf under the same conditions and concentration reduced viability by 50 ± 6%, suggesting that the complexes are significantly more toxic than Aβ aggregates alone at equal concentrations (Fig. 1B).

An increase in intracellular calcium has been observed in Aβ treated neurons as well as during glutamate excitotoxicity [18]. Therefore, we characterized calcium homeostasis after exposure of hippocampal neurons to Aβ-AChEf. Neurons were loaded for 30 min with Fluo-3 AM and then cytosolic calcium levels were evaluated in cells exposed to 5 nM AChE (Fig. 1C, silver circles), 5 μM Aβf (Fig. 1C, open circles) and 5 μM Aβ-AChEf (Fig. 1C, closed circles). Neurons treated with Aβf and Aβ-AChEf showed a marked and consistent calcium increase, while treatment with AChE enzyme alone showed no effect on the mobilization of intracellular calcium. However, the final fluorescence levels reached after 1 h treatment with Aβ-AChEf were significantly higher than Aβf, indicating that Aβ-AChEf complexes induces a sustained higher increase in intracellular calcium, in comparison with Aβ-treated neurons (Fig. 1C, inset). Then, we evaluated whether the calcium increase observed by treatments trigger the apoptotic cascade after 1 h treatment. The caspase-3 activity measurements showed that Aβ-AChEf significant increase the caspase-3 activity compared with Aβf or Aβo. On the other hand, AChE treatment did not show any effect (Fig. 1D). These results indicate that Aβ-AChE complexes showed a greater modification of intracellular calcium homeostasis than Aβ alone triggering the activation of the apoptotic pathway.

### Aβ-AChE complexes induce neurotoxicity by alteration of intracellular calcium

**Figure 2:**
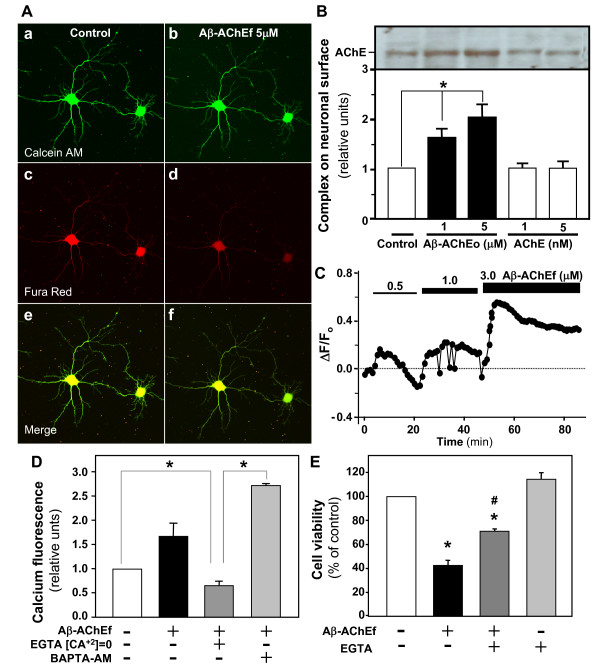
**Role of the intracellularcalcium increase in the toxicity of Aβ-AChE complexes**. (A) Hippocampal neurons were loaded with Calcein-AM to analyze the neuronal integrity and with FuraRed as an indicator of intracellular free calcium. The confocal photographs correspond to neurons before treatment (a, c and e) and after 1 h treatment with 5 μM Aβ-AChEf (b, d and f). (B) The figure shows a representative SDS-PAGE of AChE immunoblot to detect Aβ-AChEo complexes on neuronal surface by biotinylation. The graph shows a quantification of two independent experiments, results are the mean ± S.E.M. (C) Hippocampal neurons were loaded with Fluo-3 AM and treated with increasing Aβ-AChEf concentrations (μM), indicated by the black bars on top of the graph. The complexes were not washed out in between increasing concentrations. Fluorescence changes were recorded at 1 min intervals and are shown as ratio ΔF/F_o_. (D) Final normalized fluorescence intensities reached at the end of experiments are expressed over the control situation for the following treatments: 5 μM Aβ-AChEf; 2 mM EGTA; 30 μM BAPTA-AM; * *p *≤ 0.001. (E) Cell viability was evaluated by MTT assay. Hippocampal neurons were treated for 24 h as follow: 5 μM Aβ-AChEf; 2 mM EGTA. Mean MTT reduction value was expressed as % of control situation; *p ≤ 0.001 compared with the control condition; #*p *≤ 0.005 compared to the Aβ-AChEf treatment.

Given the effect of the Aβ-AChE on intracellular calcium homeostasis, we examined whether the Aβ-AChEf alter the integrity of the plasma membrane. We treated hippocampal neurons with 5 μM Aβ-AChEf and then loaded with calcein-AM and Fura-red. After 1 h treatment the fluorescence of Fura-red in neurons treated with Aβ-AChEf (Fig. 2Ad) decreased respect to the control (Fig. 2Ac), whereas the calcein fluorescence was not affected respect to the control (Fig. 2Aa and 2b), suggesting that the Aβ-AChEf treatment did not alters the integrity of the plasma membrane. We also evaluated whether Aβ-AChEo preparation interacts with the neuronal membrane through surface biotinyl assay. Fig. 2B shows that Aβ-AChEo interacts with the cell membrane, suggesting that the effects observed are because the complex is acting at the neuronal membrane level. Then, we evaluated the effect of different concentrations of Aβ-AChEf and the source of calcium responsible for the intracellular calcium increase. We determined the behaviour of calcium levels in neurons loaded with Fluo-3 AM and then treated with increasing Aβ-AChEf concentrations. We observed that the increase in the cytoplasmic calcium levels correlated with increasing Aβ-AChEf concentrations (Fig. 2C). In order to determine the source of the intracellular calcium increase induced by the complex, we treated neurons with the complex (5 μM Aβ-AChEf) in the presence of the extracellular calcium chelator, EGTA (2 mM), or the intracellular calcium chelator, BAPTA-AM (30 μM). We observed that treatment with EGTA prevented the increase of intracellular calcium induced by the complex (Fig. 2D). Additionally in neurons pre-incubated with BAPTA-AM and treated with the complex, we observed a ~ 2.8-fold increase in the intracellular calcium (Fig. 2D). These results suggest that intracellular calcium deregulation induced by the complex is dependent on extracellular calcium and that massive entrance of the ion could release intracellular calcium reservoirs. In order to study the role of the intracellular calcium deregulation in the neurotoxic properties of Aβ-AChE, we performed MTT cell viability assays in the presence of 2 mM EGTA, as indicated in Fig. 2E. Five μM Aβ-AChEf induced ~ 55% cell death, while EGTA partially reduced neuronal death triggered by the Aβ-AChEf complex (~ 25% reduction of viability), suggesting that extracellular calcium has an important role, but is not the only player in the mechanism of toxicity induced by the complex.

### Aβ-AChE complexes induce mitochondrial membrane potential loss in hippocampal neurons

**Figure 3:**
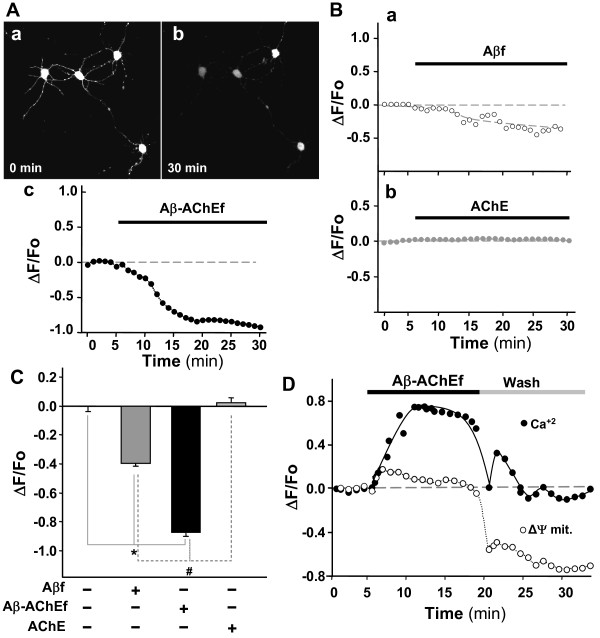
**Mitochondrial potential loss produced by Aβ-AChE complexes**. Hippocampal neurons were loaded with TMRM^+ ^to measure changes in ΔΨ_mit_. Fluorescence changes were recorded at 1 min intervals and are shown as ratio ΔF/F_o_. (A) Representative photographs of neurons a time 0 (a) or after 30 min (b) of treatment with 5 μM Aβ-AChEf, in (c), the temporal quantification of the mitochondrial potential as indicated by the black bar on top of the graph. (B) Neurons treated with 5 μM Aβf (a) or 5 nM AChE (b). (C) Normalized fluorescence intensities at the end of each treatment are expressed over the control as follow: 5 μM Aβ; 5 μM Aβ-AChEf; 5 nM AChE; * and # *p *≤ 0.001. (D) Neurons were loaded with Fluo-3 AM (black circle, 5 μM) and TMRM^+ ^(white circle, 30 nM) to measure changes in the mitochondrial membrane potential (ΔΨ_mit_). After a 5 min control period, 5 μM Aβ-AChEf was added (indicated by the black bar on top of the graph) followed by a wash with fresh recording media.

To determine whether the cytosolic calcium dysregulation induced by Aβ-AChE complexes alter mitochondrial function; we analyzed the variations in mitochondrial membrane potential (ΔΨ_mit_) of hippocampal neurons treated with the Aβ-AChEf complex, AChE and Aβf, respectively (Fig. 3). *In vivo *confocal images of hippocampal neurons exposed to Aβ-AChEf showed a severe loss of mitochondrial membrane potential using TMRM^+^, a mitochondrial potential indicator which detects alterations in mitochondrial membrane polarity (Fig. 3A) [19,20]. Photographs registered at 30 min showed a large decrease in the fluorescence of the mitochondrial potential indicator after addition of 5 μM Aβ-AChEf when compared to the beginning of the experiment (Fig. 3Aa, b). Quantification of the reduction in the mitochondria membrane potential shows a decrease of -1 normalized fluorescence unit when compared to start levels (Fig. 3A, c). Treatment with Aβf (5 μM) alone induced partial disturbances in ΔΨ_mit _levels represented by a decrease of ~ **-**0.4 normalized fluorescence units (Fig. 3B, a) and AChE treatment (5 nM) exhibited no alteration of ΔΨ_mit _levels (Fig. 3B, b). Fig. 3C shows the quantification of mitochondrial potential levels reached at the end of the experiment (1 h) in each condition. Analysis of three independent experiments showed that Aβ-AChEf induced a significant decrease in ΔΨ_mit _that was 2-fold higher than the decrease induced by Aβf treatment, while AChE had no effect on ΔΨ_mit _(Fig. 3C). To evaluate the reversibility of the effect produced by Aβ-AChEf treatment on calcium homeostasis and ΔΨ_mit _levels, hippocampal neurons were loaded with Fluo-3 AM and TMRM^+^, followed by the addition of 5 μM Aβ-AChEf for 15 min, and then washed with fresh KRH-glucose buffer. In these studies, the Aβ-AChEf induced calcium increase was completely reversed to basal levels (Fig. 3D, closed circles), however, mitochondrial membrane potential loss was not recovered after washing out of Aβ-AChEf (Fig. 3D, open circles). These results suggest that Aβ-AChE complexes could be acting at the neuronal surface, triggering an entrance of calcium depending on the presence of the complex; however, this intracellular calcium deregulation produces an irreversible effect on the mitochondrial membrane potential that could be responsible for the higher toxicity levels presented by Aβ-AChE.

### Role of mitochondria in the intracellular calcium influx induced by Aβ-AChE complexes in hippocampal neurons

**Figure 4:**
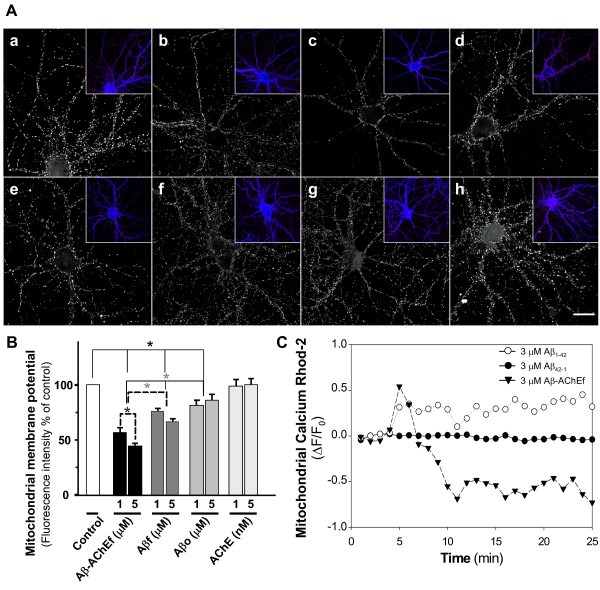
**Role of mitochondria in the calcium increase induced by Aβ-AChE**. (A) Hippocampal neurons were treated with (a) control medium, (b) 2 μM Aβ-AChEf, (c) 5 μM Aβ-AChEf, (d) 2 μM Aβf, (e) 5 μM Aβf, (f) 2 μM Aβo, (g) 5 μM Aβo and (h) 5 nM AChE for 1 h and stained with MitoTracker (red stain in the insets) and Phalloidin (blue stain in the inset) to observe neuronal morphology. Bar = 10 μm. (B) Quantification of MitoTracker fluorescence intensity representative of mitochondrial membrane potential of the different treatments. Results are the mean ± S.E.M, in duplicate experiments, n = 2 independent experiments; *p < 0.05 (C) Hippocampal neurons were loaded with 5 μM Rhod-2 AM for 40 min and mitochondrial calcium uptake were determined. Mitotracker Green™ (MTG) was used to estimate Rhod-2 signal in the mitochondria. Treatment with 3 μM Aβf gradually increased mitochondrial calcium uptake. Control experiments with reverse peptide Aβ_42-1 _did not show significant changes in mitochondrial calcium levels. However, 3 μM Aβ-AChEf complexes induced a rapid and acute mitochondrial calcium increase, with a subsequent decrease in mitochondrial calcium levels.

Treatment with Aβ-AChE complexes caused a calcium influx in hippocampal neurons and a severe reduction in mitochondrial membrane potential. In order to study the role of mitochondria in the calcium deregulation induced by Aβ-AChE, we treated hippocampal neurons with Aβ and Aβ-AChE for 1 h and stained with MitoTracker. In control neurons without treatment we observed that most of the active mitochondria were localized through neurites (Fig. 4Aa). In the case of neurons treated with Aβ-AChEf, the fluorescence of active mitochondria decreased proportional to increasing Aβ-AChEf concentrations (Fig. 4Ab, c). Aβf (Fig. 4Ad, e) reduced the fluorescence of active mitochondria more than Aβo (Fig. 4Af, g), however both showed lower effect than Aβ-AChEf (Fig. 4B). AChE treatment had no effect (Fig. 4Ah). The quantification of MitoTracker fluorescence intensity indicated that Aβf and Aβo significant decreased the mitochondrial potential respect to control neurons. However, Aβ-AChEf treatment produced around 50% decrease in the mitochondrial potential. Then, we evaluated the mitochondrial calcium uptake [20,21]. Hippocampal neurons were loaded with Rhod-2 and then treated with Aβf and Aβ-AChEf. Aβf treatment produced an increase in the calcium uptake whereas Aβ-AChEf induced an acute mitochondrial calcium increase, with a subsequent decrease in mitochondrial calcium levels (Fig. 4C). Interestingly, decrease in mitochondrial calcium uptake correlates with severe mitochondrial potential loss induced by Aβ-AChE complexes in neurons. These results suggest that Aβ-AChEf produces the release of mitochondrial calcium, according with our previous result with the intracellular calcium chelator BAPTA-AM (Fig. 2D).

### Wnt-7a, lithium chloride and a glutamate receptor antagonist, prevent the Aβ-AChE induced neuronal calcium deregulation in hippocampal neurons

**Figure 5:**
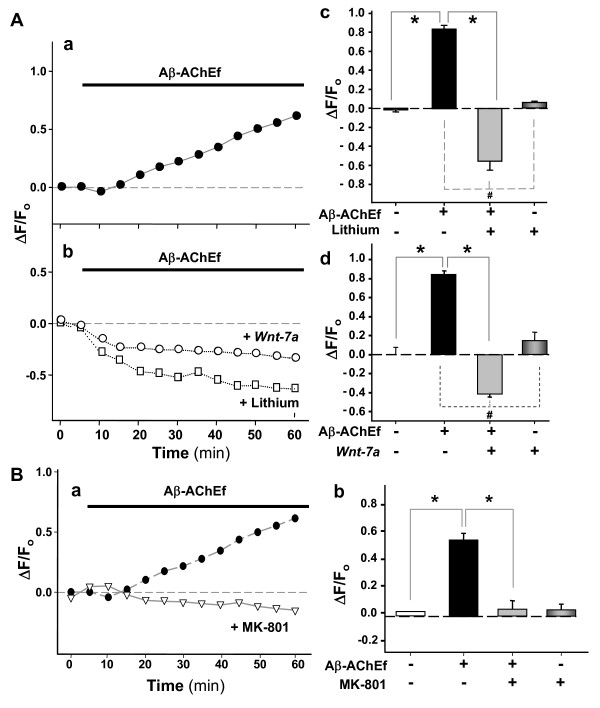
**Lithium chloride, MK-801, and Wnt-7a prevent the calcium increase induced by the Aβ-AChE complexes**. Hippocampal neurons were loaded with Fluo-3 AM and set for imaging *in vivo *experiments. Fluorescence changes were recorded at 1 min intervals and presented as the ratio ΔF/F_o_. (A) Neurons were treated with 5 μM Aβ-AChEf alone (black circle) (a), or co-incubated with 10 mM LiCl (white square), or neurons were pre-incubated with *Wnt-7a *for 24 h before AChE-Aβ treatment (white circle) (b). Final normalized fluorescence intensities reached at the end of 1 h experiments are presented and expressed over the control situation using the following treatments: 5 μM Aβ-AChEf; 5 μM Aβ-AChEf with 10 mM LiCl (c); *Wnt-7a *pre-incubation for 24 h before beginning the *in vivo *experiments (d); *p = 0.001 compared to control; #p = 0.001 compared to Aβ-AChEf treatment. (B) Hippocampal neurons were treated with 5 μM Aβ-AChEf in the presence of 2 μM MK-801 during 1 h (a), and cytoplasmic calcium levels were detected by confocal microscopy. Quantification of three independent experiments after 1 h incubation (b); *p = 0.001 compared to Aβ-AChEf treatment.

Previous studies in our laboratory showed that lithium and *Wnt *ligands protect neurons from Aβ toxicity by activation of the *Wnt *signaling pathway [22]. This pathway is also implicated in the development of the nervous system, and recently it has been demonstrated to regulate synaptic differentiation and neurotransmission [23,24]. We performed calcium experiments using Fluo-3 AM in neurons treated with Aβ-AChEf alone (Fig. 5Aa) or co-incubated with lithium or neurons pre-treated with *Wnt-7a *ligand conditioned-medium for 24 h and then exposed to Aβ-AChEf (Fig. 5Ab). Lithium (10 mM) prevented the intracellular calcium influx induced by the complex (Fig. 5Ab, open squares). In addition, *Wnt-7a *ligand also prevented the cytoplasmic calcium increase induced by the complex in hippocampal neurons (Fig. 5Ab, open circles). The quantification of the changes in fluorescence levels at 30 min into the experiment showed a significant prevention in the calcium increase triggered by Aβ-AChEf by the presence of lithium (Fig. 5Ac) and *Wnt-7a *ligand (Fig. 5Ad). Additionally, we tested the role of the NMDA receptor in the intracellular calcium deregulation induced by the Aβ-AChE complexes. In these studies, we incubated hippocampal neurons with 5 μM Aβ-AChEf in the presence of NMDA receptor antagonist MK-801 and cytoplasmic calcium changes were monitored (Fig. 5B). The inhibition of NMDA receptor activity blocked cytoplasmic calcium influx produced by the complex, (Fig. 5Ba) and the quantitative analysis of three independent experiments at 30 min into the experiments, corroborating the complete inhibition of calcium influx induced by Aβ-AChEf in hippocampal neurons (Fig. 5Bb). Additionally, inhibition of NMDA receptors prevented cytosolic calcium increase induced by 5 μM Aβ treatment (data not shown). These results suggest that both the activation of *Wnt *pathway and the inhibition of NMDA receptor prevent the cytoplasmic calcium deregulation induced by Aβ-AChE complexes in hippocampal neurons.

### Presence of AChE increases Aβ oligomer formation and neurotoxicity in hippocampal neurons

**Figure 6:**
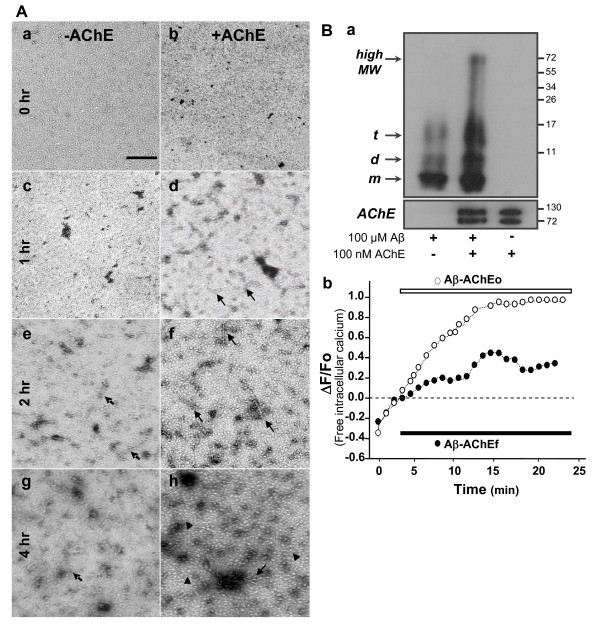
**Effect of Aβ-AChE oligomers in hippocampal neurons**. (A) Aβ (5 μM) in the absence (a, c, e, g) and presence of 50 nM AChE (b, d, f, h) was aggregated at 37°C without stirring. 5 μl aliquot was obtained at 0 (a, b); 1 (c, d); 2 (e, f) and 4 hr (g, h), stained with 2% uranyl acetate and photographed with an electron microscope. More Aβ oligomers were formed in the presence of AChE, corresponding to protofibrils (arrow) (f), or amylospheroids (head-arrow) (h). Scale bar, 100 nm. (B) Western blot for 50 μM Aβ aggregated alone or in the presence of 50 nM AChE, an aliquot of supernatant obtained after centrifugation was used (a). Hipoccampal neurons in culture were loaded with Fluo-3 AM (5 μM for 20 min at 37°C) to measure changes in free intracellular Ca^2+ ^every 1 min. The graphic shows normalized fluorescence intensities according to the ratio ΔF/F_o _(arbitrary units) in function of time. Black bar indicates onset of treatment. 5 μM Aβ-AChEf (black circle); 5 μM Aβ-AChEo (white circle) (b).

It has been suggested that Aβ oligomers are responsible for the toxic effects of Aβ [25-27]. Additionally, Aβ oligomers induced an accelerated calcium influx, mitochondrial depolarization, decreased ATP levels, and cytochrome c release in cortical neurons [28]. Therefore, we decided to evaluate whether Aβ-AChE containing Aβ oligomers, show the same toxicity found in hippocampal neurons exposed to the complex containing Aβ fibrils. Aβ was aggregated, at 37°C, in the absence or presence of AChE enzyme and aliquots were taken at different times of Aβ aggregation and analyzed by electron microscopy. The formation of Aβ oligomers was faster in the presence of enzyme AChE (compare picture Fig. 6Ac with 6Ad). The microphotographs show oligomeric structures present at 1 h of AChE and Aβ incubation (Fig. 6Ad also, Additional file 1), where in the case of Aβ alone, oligomeric structures appear only after 2 h incubation (Fig. 6Ae). Clearly, there are more Aβ oligomers formed in the presence of AChE, which correspond to either protofibrils (Fig. 6Af arrow), or amylospheroid assemblies (Fig. 6Ah, arrow head). Previous studies indicated that high Aβ oligomer neurotoxicity is associated with an accelerated calcium influx and mitochondrial impairment [28]. In addition, Aβ oligomers prepared in the presence of AChE were subjected to non-denaturing electrophoresis gels and western blotting for Aβ (Fig. 6Ba). The western blot showed 3 bands corresponding to monomers, dimmers and trimmers of Aβ when aggregation was performed without the presence of AChE enzyme (Fig. 6Ba, line 1). When Aβ was incubated with AChE, we observed the 3 bands for monomers, dimmers and trimmers plus a high molecular weight band of 74 KDa (Fig. 6Ba, line 2). In order to see whether AChE-Aβo affect intracellular Ca^2+^, hippocampal neurons were loaded with Fluo-3 AM. The use of an oligomeric-rich preparation (Aβ-AChEo) induced a faster and prominent calcium increase (~ 0.6 normalized fluorescence units during the first 10 min of treatment) respect to neurons treated with normal Aβ-AChEf complexes (~ 0.2 normalized fluorescence units during the first 10 min of treatment), indicating that the oligomeric-rich preparation had a fast effect on calcium homeostasis (Fig. 6Bb). These results are in agreement with the evidence that indicates that Aβ oligomers induce a similar effect on cell viability and calcium homeostasis in a human neuroblastoma cell line [28].

## Discussion

AChE is an enzyme involved in diverse functions in both central and peripheral nervous systems and it has been demonstrated to be associated with senile plaques [3]. On the other hand, a renewed interest in calcium as part of the pathology of AD began with the recent observation of polymorphism in a CALHM1 gene that may influence calcium mutations and increase the risk of AD [29]. Indeed, several studies indicate that familial AD mutations in presenilins affect calcium homeostasis, releasing intracellular calcium from ryanodine or IP3 channels [30].

We had reported here that Aβ-AChE complex trigger more neurodegeneration than the Aβ alone, acting as a chaperone protein which increases the toxicity of Aβ peptide, confirming previous studies both *in vitro *and *in vivo *[9,10]. We have shown that a short exposure of hippocampal neurons to Aβ-AChE complexes caused an influx of calcium with higher levels than the amyloid fibrils alone and this increase was reversible and concentration-dependent; in addition the calcium source for such increase was the extracellular calcium present in the media, which probably release some intracellular calcium reservoirs, as mitochondria. Our results suggest that Aβ-AChE complexes are exerting their actions at the plasma membrane level, possibly acting on a calcium channel or on some yet unidentified cell surface receptor-channel that could activate calcium entry upon Aβ complex interaction [14]. Disruption of intracellular homeostasis of Ca^2+ ^and K^+ ^by opening of these channels, has been extensively proposed as a mechanism of Aβ neurotoxicity [14,18]. It has been reported that Aβ-induced activation of AChE in P19 cells was mediated through the opening of L-type calcium channels [31]. Also, it has been found that Aβ induces production of an amphiphilic monomeric isoform of AChE through a mechanism that involves at L-type calcium channels. This specific increase in a minor isoform of the enzyme has also been seen to be increasing in transgenic mice that overexpress AβPP [32], as well as in the brains of rats that received intracerebral injections of Aβ peptides [33]. It is also increased in the brain and cerebrospinal fluid of patients with AD [34]. These studies suggest an active role of the Aβ-AChE complexes in the AD pathology. Aβ-AChE complexes and Aβ treatment had different effects over the mitochondrial membrane potential (ΔΨ_mit_) observed with the TMRM^+ ^dye. Our experiments indicated that Aβ-AChE complexes affected ΔΨ_mit _more than Aβ; also, we observed that the mitochondrial membrane potential was compromised in a non-reversible manner, even when the calcium increase was reverted after wash out. These events could be related with previously described mitochondrial dysfunction effects, which included the mitochondria Permeability Transition Pore (mPTP) opening [35]. This structure is involved in cytochrome c release, apoptotic mechanisms on high levels of cytosolic calcium and oxidative stress excess contribute to this structure opening and finally affect mitochondrial membrane potential, ATP levels, and mitochondrial calcium uptake as we observed in Fig. 4C[35-37]. Our results indicate that the increased neurotoxicity of Aβ-AChE complexes with regard to Aβ aggregates is probably related to a more rapid and non-reversible increase of mitochondrial dysfunction or to some direct effects at the mitochondrial viability level.

It has been described that Aβ oligomers increase intracellular calcium [28]. We showed that Aβ in the presence of AChE produces Aβ oligomers faster than Aβ aggregates incubated without the enzyme, an effect that could explain why the alterations triggered by Aβ-AChE complexes were stronger at Ca^2+ ^homeostasis level.

Previous studies from our laboratory indicated that lithium protects hippocampal neurons against Aβ peptide and Aβ-AChE complex neurotoxicity [22,38]. Additionally, we found that pre-incubation with *Wnt-7a *ligand prevents the increase in cytosolic calcium induced by Aβ [39]. Hence, in order to study whether *Wnt *signaling had any effect over the observed calcium increase, we performed time-lapse experiments co-treating neurons with Aβ-AChE complexes in the presence of *Wnt-7a *or lithium. These experiments proved that *Wnt-7a *and lithium prevent the cytoplasmic calcium influx induced by these complexes. These results are also consistent with the idea that lithium induces NMDA receptor inactivation with a concomitant decrease in the cytosolic calcium increase induced by glutamate [40]. Moreover, lithium treatment reduced the level of NR2B subunit phosphorylation at Tyr1472 which results in inactivation of NMDA receptors contributing to neuroprotection against glutamate excitotoxicity [40]. Furthermore, it has been reported that lithium robustly protected individual brain mitochondria loaded with Rhodamine 123 (mitochondrial potential dye) against Ca^2+^-induced depolarization, an event that was mediated by inhibition of mPTP activation by lithium [41]. Taken together, this evidence and our studies allow us to suggest a novel and interesting role of lithium protection against mitochondrial dysfunction triggered by calcium dyshomeostasis.

Excessive glutamate, an abundant neurotransmitter in the brain, is toxic to neurons and can lead to intracellular calcium overload and neuron death. Overstimulation of the glutamatergic system, also known as glutamate excitotoxicity is observed in a number of neurodegenerative diseases including AD [42]. In fact, memantine, an open-channels blocker of the NMDA receptor is use to treat moderate and severe AD [43].

We have studied here, the role of the NMDA receptor in neurotoxic effects induced by Aβ-AChE complexes using MK-801, a glutamate receptor antagonist, our studies indicates that the NMDA receptor antagonist, totally prevents the cytoplasmic calcium influx induced by the complex. Therefore, it may be possible that the decrease observed in calcium cytosolic levels may be due in part to inactivation of the NMDA receptors by lithium. Further studies are necessary to clarify this issue.

## Conclusions

We report here new findings about the Aβ-AChE complex mechanism, which includes a massive cytoplasmic calcium influx and severe mitochondrial dysfunction in rat hippocampal neurons. The pathologic entry of calcium determines that mitochondria participate in the buffering of cation excess, and this process leading to its membrane depolarization and loss of viability. The increased Aβ-AChE complexes neurotoxicity observed with regard to Aβ aggregates could be explained by a rapid loss of the mitochondrial membrane potential. These events may be related to key neuropathological mechanism of AD, and Aβ-AChE complexes might cause further damage through changes in calcium homeostasis and mitochondrial function, as well as at other neuronal levels.

## Methods

### Reagents

Synthetic Aβ_1-42 _and Aβ_42-1 _peptides corresponding to the human Aβ wild-type and reverse sequence, respectively and both were obtained from Genemed Synthesis, Inc. (San Francisco, CA). Chemicals, culture media and sera were obtained from Sigma (St. Louis, MO), Roche (Alameda, CA), Merck (Darmstadt, Germany), Gibco BRL (Paisley, UK). Fluo-3 AM, Calcein-AM, Fura Red AM, and Bapta AM from Molecular Probes (Eugene, OR). Rhod-2AM, and TMRM^+ ^(Xanthylium,3,6-bis(dimethylamino)-9-(2-(methoxycarbonyl)phenyl)-, perchlorate) were a kind gift of Dr. Luis Felipe Barros (CECS, Valdivia, Chile).

### AChE Purification

Tetrameric G_4 _AChE form (sedimentation coefficient, 10.7 S) was purified from bovine caudate nucleus, using acridine affinity chromatography as previously described [44]. Both specific activity (6,000 U/mg protein) and staining intensity after sodium dodecyl sulfate-polyacrylamide gel electrophoresis were used to verify purity.

### Formation of Amyloid Species

Aβ aggregates were formed in a turbidity assay as previously described [45]. Briefly, For Aβf and Aβ-AChEf, the Aβ peptide stock solution was prepared by dissolving freeze-dried aliquots of Aβ in dimethyl sulfoxide (DMSO) at 15 mg/ml (3.5 mmol/L). An aliquot of this stock solution equivalent to 15 μg of Aβ peptide was added to aqueous buffer (725 μl total volume; 100 μM PSB, pH 7.4). For the aggregation assay in the presence of AChE, an identical aliquot of the stock solution was added to a buffer containing AChE (100 nM). The solutions were stirred continuously (1,350 rpm) at room temperature for 24 h and then left at 4°C for another 48 h, then an aliquot was taken and observed by electron microscopy. Aggregation was measured by turbidity at 405 nm against a buffer blank. Amyloid aggregates obtained were characterized by Thioflavin-T (Th-T) binding [45].

### Preparation of Aβ oligomers

Aβoligomers were formed using Aβ peptide in the presence of AChE. Briefly, a 50 μM Aβ stock solution was prepared in the presence of 50 nM AChE. The solution was kept at 37°C for 1 h and then used immediately. An aliquot of Aβo and Aβ-AChEo was separated by centrifugation at 14,000 rpm (15 min) for electron microscopy. The supernatant was saved for non-denaturing SDS-PAGE gels using SDS-free and β-mercaptoethanol-free loading buffer and then regular western blot.

### Electron microscopy

Fresh aliquots of samples were diluted 1:3 in water and 5 βl placed on Parlodion/carbon-coated 300-mesh copper grids for 60s. Excess sample was removed and 5 μl of 2% aqueous uranyl acetate was placed onto the grid for 10s, followed by removal of excess staining solution with filter paper and air-drying. Observations were carried out using a Philips Tecnai 12 electron microscope operated at 80 kV. Photographs were taken at original magnifications of 49,000×. Copies from the negatives were made with a further 3× magnification of given areas and used for figure presentation.

### Culture of rat hippocampal neurons

Hippocampi from Sprague-Dawley rats at embryonic day 18 were dissected and primary rat hippocampal cultures were prepared as described previously [39,45]. Hippocampal cells were seeded in polylysine-coated wells and cultivated in Neurobasal medium supplemented with B27, on day 3 of culture; cells were treated with 2 μM 1-β-D-arabinofuranosylcytosine (AraC) for 24 h to reduce most of the glial cells present in the culture. Seven days later, cultured hippocampal neurons were used for various experiments. The average number of neurons in each experiment corresponded approximately to 98% of total cells present in the cultures.

### Cell survival assay

Cell viability was measured by the modified 3-[4,5-dimethylthiazol-2yl]-2,5-diphenyltetrazolium bromide (MTT) assay as described previously [45].

### Immunofluorescence staining

Hippocampal neurons plated on polylysine-coated covers (25,000 cells/cover) were treated and then fixed with 4% paraformaldehyde/4% sucrose in PBS for 20 min, permeabilized with 0.2% Triton-X100 for 5 min, blocked with 0.2% gelatine and immunostained using an anti- Map-1B, neurofilament protein, synapsin-1 or PSD-95 antibody (1:500). Coverslips were mounted and then analyzed under a Zeiss confocal microscope.

### Morphometric analysis

The number and length of neurites were quantified using an Image-Pro plus software as described previously [38].

### Caspase-3 Activity

Hippocampal neurons 15 DIV were treated with Aβ-AChEf, Aβo and Aβf for 1 h at 37°C, then the cells were homogenized with RIPA buffer and then lysates were centrifuged at 15,000 g for 20 min at 4°C, the supernatant were collected to determinate Caspase activity. Caspase-3, substrate chromogenic (Upstate Biotechnology Inc., Lake Placid, NY, USA) was prepared in 12.5 mM buffer Hepes; 31.25 w/v Sucrose; 0,3125 w/v CHAPS pH 7.4. The reaction was measured at 405 nm in a microtiter plate reader.

### Aβ-AChE in plasmatic membrane

Hippocampal neurons 15 DIV were treated with Aβ-AChEo (1 or 5 μM) or AChE alone (1 or 5 nM) for 1 h and membrane surface proteins were biotinylated with sulfo-NHS-LC-biotin (Pierce) in a final concentration of 0.5 mg/mL for 45 min. After biotinylation step, the free biotin was quenched by incubation with 50 mM NH_4_Cl for 10 min. Cells were lysed in ice-cold SA buffer (150 mM NaCl; 20 mM Tris pH 8.0; 5 mM EDTA; 1% Triton-X-100; 0.2% BSA and protease inhibitors). Nuclear and cellular debris was removed by centrifugation at 14,000 x g for 5 min at 4 °C and the biotinylated cell-surface proteins were then adsorbed to streptavidin agarose beads for 16 h at 4 °C. Beads were washed and bound proteins were analyzed by SDS-PAGE followed by immunoblotting against AChE. The values for biotinylated cargo proteins were normalized to total cargo proteins expressed in the cells.

### Mitochondria membrane potential

Changes in mitochondrial membrane potential was determined by specific mitochondrial probe TMRM^+ ^and detected in a confocal microscope [19,46]. Neurons were grown on poly-L-lysine-coated glass coverslips and cultured for 5 days. The cells were then loaded for 30 min with 30 nM TMRM^+ ^in Krebs-Ringer-Hepes (136 mM NaCl; 20 mM Hepes; 4.7 mM KCl; 1.25 mM MgSO_4_; 1.25 mM CaCl_2_) (KRH)-glucose containing 0.02% pluronic acid, then washed, and allowed to equilibrate for 30 min. Coverslips were then mounted in a chamber on the stage of a confocal laser scanning microscope (LSM Pascal Zeiss model 510, Carl Zeiss Ltd.). The fluorescence changes determined by TMRM fluorescence indicated the mitochondria potential changes. Images were acquired using a 543-nm He-Ne laser to excite TMRM^+ ^and the signals were collected at 570 nm [18-20]. Signal from control neurons and neurons treated with Aβ-AChE complexes were compared using identical settings for laser power, confocal thickness and detector sensitivity. The images were analyzed with Zeiss confocal software, the mean TMRM^+ ^fluorescence signal was measured per live cell. Estimation of fluorescence intensity of TMRM^+ ^was presented like the pseudoratio (ΔF/F_o_) indicated by: ΔF/F_o _= (F-F_base_)/(F_base_-B), where F is the measured fluorescence intensity of the indicator, F_base _is the fluorescence intensity before the stimulation, and B is the background signal determined from the average of areas adjacent to the cells. For mitochondrial membrane potential by MitoTracker fluorescence, hippocampal neurons 15 DIV were treated with Aβ-AChEf, Aβo and Aβf for 1 h at 37°C. Then, cells were stained with MitoTrackers-fx (Molecular Probes, Eugene, OR). In all treatments, individual coverslips were rinsed twice in PBS and loaded with 10-500 nM MitoTracker for 15 min at 37°C. Dyes were diluted in PBS from a 1 mM stock in anhydrous dimethyl sulfoxide. Coverslips were rinsed for 15 min in PBS at room temperature. Loading parameters were tested and optimized for assessing fluorescence intensity and localization in Confocal Zeis Microscope and the quantification was made using Image J Program.

### Cytoplasmic Calcium Imaging

Neurons grown on glass coverslip were loaded for 30 min (37°C) with the following fluorescent probes as their acetoxymethyl (AM) ester forms: 5 μM Fluo-3 AM and 10 μM Rhod-2 AM in Krebs-Ringer-Hepes (KRH)-glucose containing 0.02% pluronic acid. The fluorescence changes determined by Fluo-3 represent the cytoplasmic calcium [Ca^2+^]_cyt _changes. Coverslips were washed three times with PBS and left in KRH-glucose for 10 min until cell fluorescence had reach plateau. Fluorescence was imaged with a confocal laser scanning microscope as described previously [19]. Images were acquired using a 488-nm Argon laser to excite Fluo-3 and Fura-Red fluorescence. The signals were collected at 505-530 nm (Fluo-3). Background was measured in parts of the field devoid of cells and found to be not significantly different from the signal recorded in cells depleted of dye with 100 μM digitonin. This value was subtracted from cell measurements. The fluorescence intensity variation was recorded from 15-20 neurons in average per experiment. Estimation of fluorescence intensity of Fluo-3 was presented like the pseudoratio (ΔF/F_o_), as indicated before [18-20].

### Cell membrane integrity

Hippocampal neurons were loaded 30 min with Calcein-AM (Molecular Probes, Leiden, Netherlands) as an indicator of cell integrity [19].

### Mitochondrial Calcium Measurements

Hippocampal neurons were grown on 35-mm dishes and loaded with 5 ìM Rhod-2 AM in KRH-glucose buffer containing 0.02% pluronic acid. The fluorescence changes determined by Rhod-2 indicate calcium changes in the mitochondria [19]. To estimate Rhod-2 fluorescence pattern in live mitochondria, we used MitoTracker Green™ (MTG) to mark the mitochondria. Cells were washed 3 times and left in KRH-glucose buffer for 10 min until cell fluorescence equilibrated. Fluorescence was imaged with a confocal laser scanning microscope (Leica TCS SP1) using a 40× water immersion lens. Images were acquired using a 488-nm argon laser to excite MTG fluorescence and a 563-nm He-Ne laser to excite Rhod-2 fluorescence. The signals were collected at 505-530 nm (MTG) and at 590 nm (Rhod-2). Fluorescence background signal was subtracted from cell fluorescence measurements in every experiment. The fluorescence intensity variation was recorded from 5-10 cells on average per experiment. Estimation of fluorescence intensities were presented as the pseudoratio (ΔF/F_o_), which was calculated using the formula ΔF/F_o _= (F - F_base_)/(F_base _- B), where F is the measured fluorescence intensity of the indicator, F_base _is the fluorescence intensity before the stimulation, and B is the background signal determined from the average of areas adjacent to the cells [21].

### Statistical analysis

Results were expressed as mean ± standard error. Student's *t *test and the Mann-Whitney test were used for analyzing data for fluorescence measurements and image analysis. P < 0.05 was regarded as statistically significant.

## Competing interests

The authors declare that they have no competing interests.

## Authors' contributions

MCD participated in the design of the experiments, carried out part of the oligomeric analysis with the Aβ and the Aβ-AChE, electron microscopy and imunofluorescence, helped in the interpretation of the results and drafted the manuscript. JPS and RAQ participated in calcium, mitochondrial and live cell experiments. MA helps in the characterization of Aβ oligomers and the Aβ-AChE complexes. JAG carried out the cell viability experiments and fixed mitochondrial membrane potential. JAG and RAQ helped to draft the manuscript. NCI design the studies and revise the manuscript. All authors read and approved the final manuscript.

## Supplementary Material

Additional file 1 **Characterization of the aggregates used for the experiments by electron microscopy**. Negative stain and electron microscopy photographs for 5 μM of (A) Aβ-AChEf and (B) Aβ-AChEo preparations used in the experiments. Click here for file

Additional file 2 **Effect of Aβ and Aβ-AChE aggregates on synaptic proteins**. Immunofluorescence assay for PSD-95 of hippocampal neurons treated with (A) control or 1 μM of (B) Aβo, (C) Aβf, (D) Aβ-AChEo, (E) Aβ-AChEf. Also a magnification of neurites in each treatment is showing. The imunofluorescence correspond to synapsin-1 (red) and PSD-95 (green). Click here for file
