# Supplementary figures and images for: Amyloid-β-Acetylcholinesterase complexes potentiate neurodegenerative changes induced by the Aβ peptide. Implications for the pathogenesis of Alzheimer's disease
Source: Mol Neurodegener. 2010 Jan 18;5:4. doi: 10.1186/1750-1326-5-4 (PMC2823746; doi:10.1186/1750-1326-5-4)

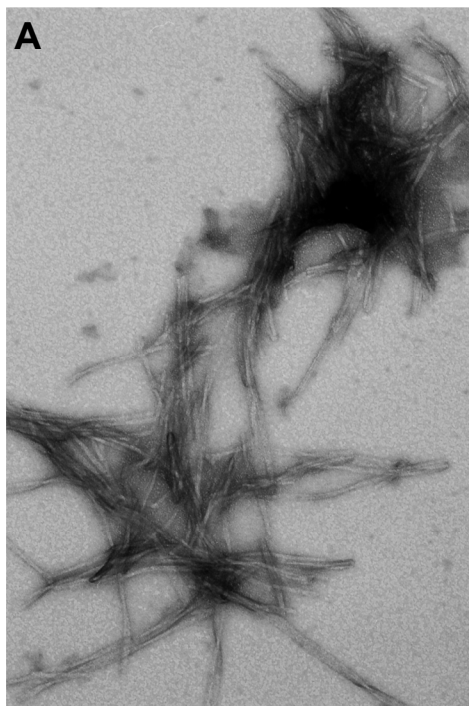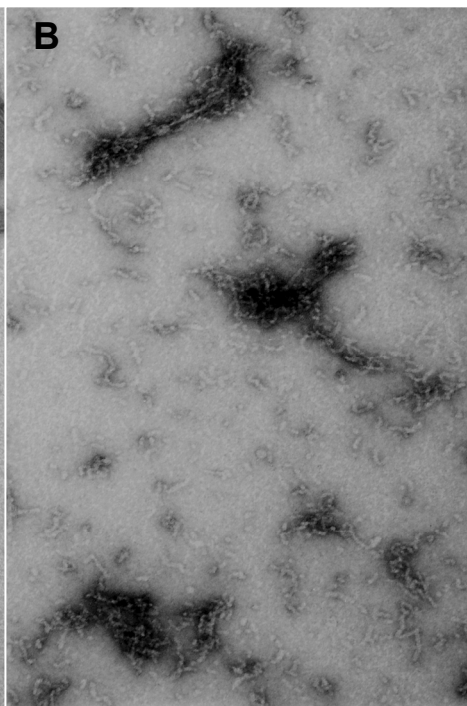

Supplement: Additional file 1 — Characterization of the aggregates used for the experiments by electron microscopy. Negative stain and electron microscopy photographs for 5 μM of (A) Aβ-AChEf and (B) Aβ-AChEo preparations used in the experiments. [file 1750-1326-5-4-S1.PDF]

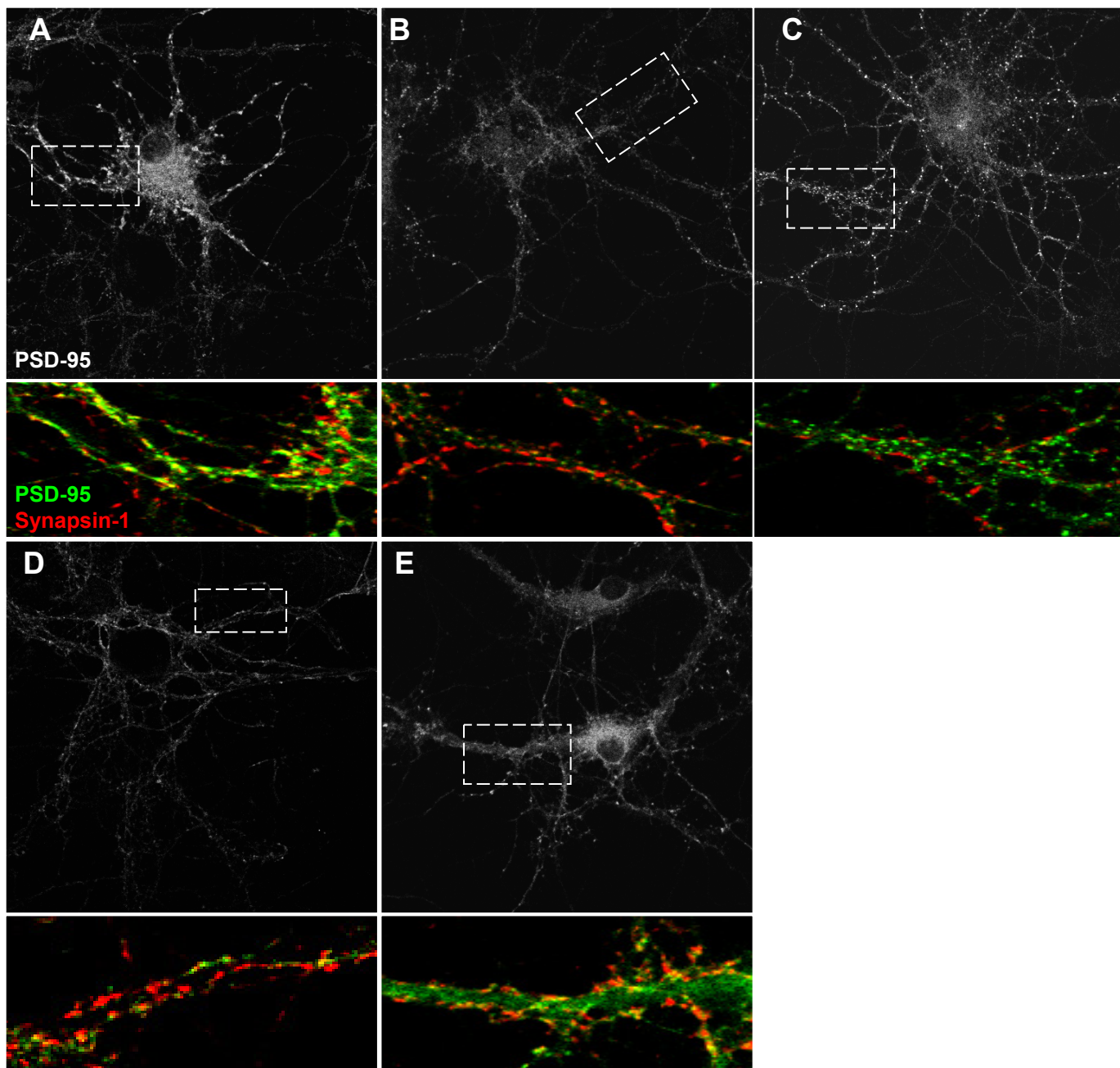

Supplement: Additional file 2 — Effect of Aβ and Aβ-AChE aggregates on synaptic proteins. Immunofluorescence assay for PSD-95 of hippocampal neurons treated with (A) control or 1 μM of (B) Aβo, (C) Aβf, (D) Aβ-AChEo, (E) Aβ-AChEf. Also a magnification of neurites in each treatment is showing. The imunofluorescence correspond to synapsin-1 (red) and PSD-95 (green). [file 1750-1326-5-4-S2.PDF]
